# Metabolic Profile and Lipid Metabolism Phenotype in Mice with Conditional Deletion of Hepatic BMAL1

**DOI:** 10.3390/ijms25116070

**Published:** 2024-05-31

**Authors:** Weijia Gu, Ting Li, Yuxin Huang, Ruiqing Wang, Lu Zhang, Rucheng Chen, Ran Li, Cuiqing Liu

**Affiliations:** 1 School of Public Health, Zhejiang Chinese Medical University, Hangzhou 310053, China; wjgu@zcmu.edu.cn (W.G.);; 2 Zhejiang International Science and Technology Cooperation Base of Air Pollution and Health, Hangzhou 310053, China

**Keywords:** circadian rhythm, BMAL1, lipid metabolism, triglyceride, mitochondrial β-oxidation

## Abstract

The disruption of circadian rhythms (CRs) has been linked to metabolic disorders, yet the role of hepatic BMAL1, a key circadian regulator, in the whole-body metabolism and the associated lipid metabolic phenotype in the liver remains unclear. *Bmal1* floxed (*Bmal1*^*f*/*f*^) and hepatocyte-specific *Bmal1* knockout (*Bmal1*^*hep−*^^/*−*^) C57BL/6J mice underwent a regular feeding regimen. Hepatic CR, lipid content, mitochondrial function, and systemic metabolism were assessed at zeitgeber time (ZT) 0 and ZT12. Relevant molecules were examined to elucidate the metabolic phenotype. Hepatocyte-specific knockout of *Bmal1* disrupted the expression of rhythmic genes in the liver. *Bmal1*^*hep−*^^/*−*^ mice exhibited decreased hepatic TG content at ZT0, primarily due to enhanced lipolysis, reduced lipogenesis, and diminished lipid uptake. The β-oxidation function of liver mitochondria decreased at both ZT0 and ZT12. Our findings on the metabolic profile and associated hepatic lipid metabolism in the absence of *Bmal1* in hepatocytes provides new insights into metabolic syndromes from the perspective of liver CR disturbances.

## 1. Introduction

The circadian rhythms (CRs), or biological clock, orchestrates recurring physiological changes over a 24-hour cycle. Governed by a central clock situated in the anterior suprachiasmatic nucleus (SCN) and peripheral clocks in various tissues [1], the peripheral clocks regulate daily gene expression for physiological processes [2,3]. The precise regulation of the biological clock involves a set of rhythmic genes known as clock-controlled genes (CCGs) [4]. CCGs, including the central gene *Bmal1* (brain and muscle arnt-like 1), form a feedback loop with *Clock*, *Per*, and *Cry*, collectively regulating the CR [5]. Disruption of the CR, as seen in global *Bmal1* knockout mice, leads to loss of rhythm, premature aging, tendon calcification, reduced locomotor activity and body weight, and elevated reactive oxygen species levels [3,6,7,8]. A well-functioning CR is crucial for proper maintenance of physiological processes.

The liver is integral to lipid metabolism, with hepatocytes intricately regulating processes such as fatty acid uptake, oxidation, synthesis, and release under the precise control of CCGs [9]. *Bmal1* plays a regulatory role in hepatic lipid synthesis, encompassing lipoprotein synthesis [10,11]. In our previously study, we found that exposure to ambient fine particulate matter (PM_2.5_) disturbed hepatic *Bmal1* circadian oscillations at ZT0 and disrupted hepatic lipid metabolism at ZT12 [12]. Its global deletion has been demonstrated to be involved in glucose homeostasis and lead to dysregulation of the Elovl3 rhythm in the liver [13], indicating an association between hepatic lipid metabolism stability and precise CR regulation. Previous studies on the impact of BMAL1 on liver metabolism have mostly focused on external zeitgebers (such as restricted feeding) [14,15] or non-alcoholic fatty liver disease (NAFLD) induced by a high-fat diet (HFD) [7,8,11]. There is a scarcity of comprehensive research on the effects of BMAL1 on liver metabolism under normal physiological conditions (unrestricted access to food and water with a standard chow diet).

This study provides the metabolic profile by employing a hepatocyte-specific *Bmal1* knockout (*Bmal1*^*hep−*^^/*−*^) mouse model and examines the specific role of clock gene *Bmal1* in hepatic lipid metabolism.

## 2. Results

### 2.1. Construction of Hepatocyte-Specific Bmal1 Knockout Mice and the CCG Profile in the Liver

**Figure 1:**
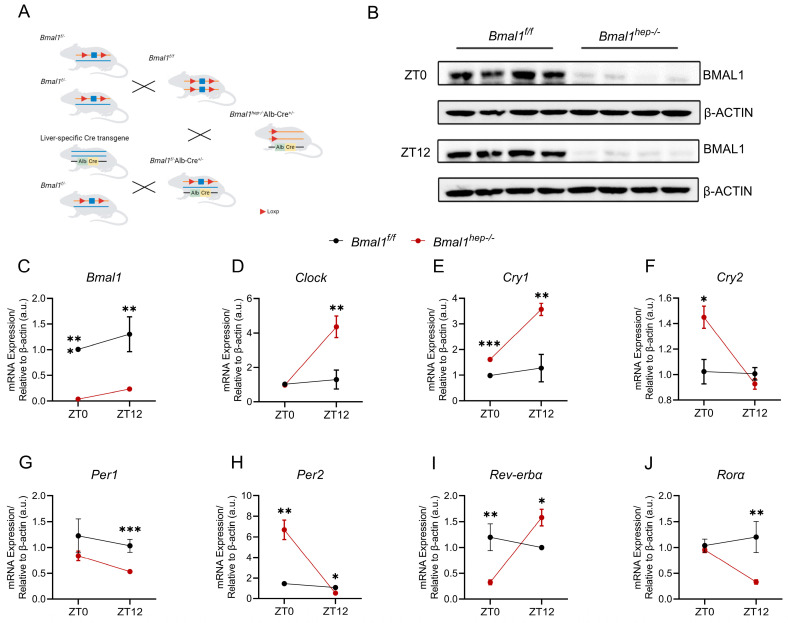
Hepatocyte-specific *Bmal1* knockout disrupted CR of hepatic CCGs. (**A**). The CRISPR/Cas9 strategy employed is depicted schematically. (**B**). Western blot analysis of BMAL1 in the liver tissue of *Bmal1*^*f*/*f*^ and *Bmal1*^*hep−*^^/*−*^ mice ZT0 and ZT12. β-Actin was used as a loading control. Quantification of the results was performed from three independent experiments. (**C**–**J**). Diurnal changes in expression of *Bmal1*, *Clock*, *Cry1*, *Cry2*, *Per1*, *Per2*, *Rev-erbα*, and *Rorα* in the liver tissue of *Bmal1*^*f*/*f*^ and *Bmal1*^*hep−*^^/*−*^ mice. The values are presented as mean ± SEM. *Bmal1*^*f*/*f*^ mice, n = 11; *Bmal1*^*hep−*^^/*−*^ mice, n = 12. * *p* < 0.05, ** *p* < 0.01, *** *p* < 0.001.

In our previously study, we found that exposure to PM_2.5_ disturbed *Bmal1* circadian oscillations at ZT0 and disrupted hepatic lipid metabolism at ZT12 [12]. To build upon our previous findings, we generated *Bmal1*^*hep−*^^/*−*^ mice by crossing *Bmal1*^*f*/*f*^ mice with Alb-Cre mice (Figure 1A). In *Bmal1*^*hep−*^^/*−*^ mice, *Bmal1* expression was markedly reduced and abolished in liver tissue (Figure 1B,C).

We examined the expression of core CCGs in the liver of mice at ZT0 and ZT12. At ZT0, *Bmal1*^*hep−*^^/*−*^ mice displayed a significant upregulation of *Cry1*, *Cry2*, and *Per2*, with a significant downregulation of *Rev-erbα*. At ZT12, *Bmal1*^*hep−*^^/*−*^ mice exhibited a significant upregulation of *Clock*, *Cry1*, and *Rev-erbα*, while *Per1*, *Per2*, and *Rorα* were significantly downregulated (Figure 1D–J). These findings collectively indicated substantial alterations in hepatic CCGs resulting from hepatocyte-specific *Bmal1* knockout.

### 2.2. Profiles of Hepatic Lipid Metabolism in Hepatocyte-Specific Bmal1 Knockout Mice

**Figure 2:**
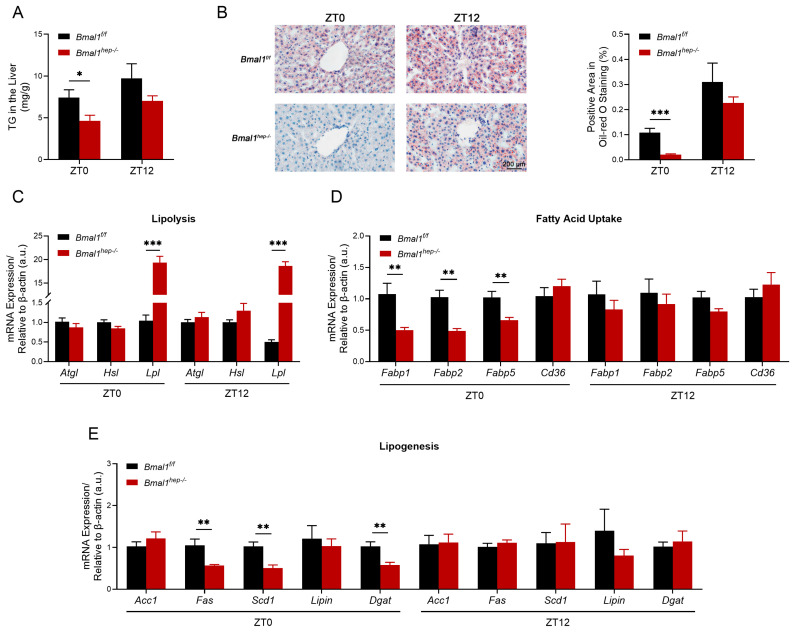
The measurement of TG in the liver and the diurnal changes of corresponding lipid metabolism enzyme genes. (**A**). Total TG levels in the hepatic lipid extracts at ZT0 and ZT12. (**B**). Representative pictures of Oil Red O staining and the corresponding area analysis at ZT0 and ZT12. The black scale bar represents 200 μm. (**C**–**E**). mRNA levels of enzymes involved in hepatic lipolysis (**C**), fatty acid uptake (**D**), and lipogenesis (**E**) at ZT0 and ZT12. *Bmal1*^*f*/*f*^ mice, n = 6; *Bmal1*^*hep−*^^/*−*^ mice, n = 6. * *p* < 0.05, ** *p* < 0.01, *** *p* < 0.001.

Liver-clock mutant mice exhibit impaired lipid homeostasis, which is partially regulated by BMAL1/LIPIN/DGAT signaling [16,17]. Combined with our previous research findings, we investigated hepatic lipid metabolism in *Bmal1*^*hep−*^^/*−*^ mice, focusing on changes in lipolysis, lipid uptake, and synthesis. At ZT0, the level of TG was significantly lower in *Bmal1*^*hep−*^^/*−*^ mice (Figure 2A), and this finding was confirmed by Oil Red O staining (Figure 2B). The expression of enzymes for lipolysis (*Lpl* but not *Atgl* or *Hsl*) significantly increased (Figure 2C), while the expression of fatty acid uptake receptors (*Fabp1*, *Fabp2*, and *Fabp5*) (Figure 2D) and lipogenic enzymes (*Fas*, *Scd1*, and *Dgat*) (Figure 2E) significantly decreased. At ZT12, the expression of *Lpl* still exhibited a significant increase in *Bmal1*^*hep−*^^/*−*^ mice (Figure 2C) with no change in the level of TG (Figure 2A,B), while other genes that previously exhibited changes at ZT0 did not show significant differences (Figure 2D,E). No significant difference was observed in lipid export genes (*Mttp* and *Apob*) between *Bmal1*^*f*/*f*^ and *Bmal1*^*hep−*^^/*−*^ mice (Supplementary Figure S1). Based on the findings, the decrease in hepatic TG levels at ZT12 in the 24th week could be attributed to heightened lipolysis, diminished lipogenesis, and decreased expression of lipid receptors resulting from the deletion of *Bmal1*.

### 2.3. Profiles of Hepatic Mitochondrial Function in Hepatocyte-Specific Bmal1 Knockout Mice

**Figure 3:**
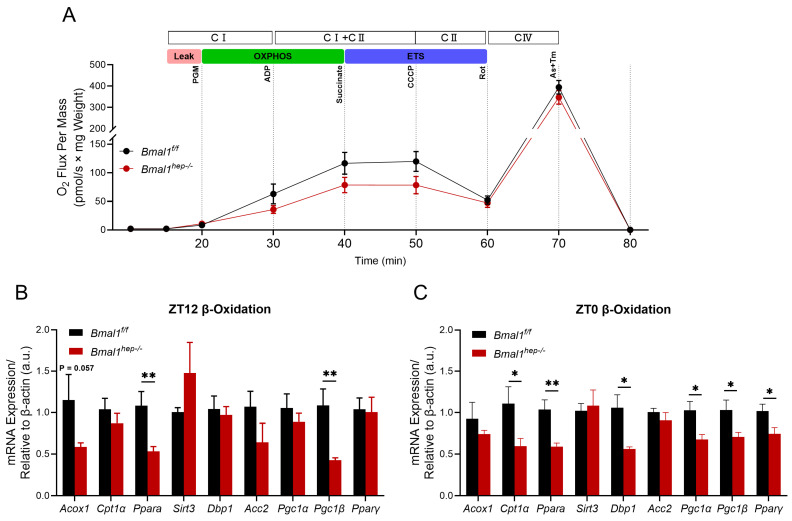
Combined determination of oxygen flux by O2k-Fluorometry in isolated hepatic mitochondria and expression of β-oxidation-related genes. (**A**). Oxygen flux changes relative to the liver weight. *Bmal1*^*f*/*f*^ mice, n = 3; *Bmal1*^*hep−*^^/*−*^ mice, n = 5. (**B**,**C**). Diurnal changes in expression of *Acox1*, *Cpt1α*, *Ppara*, *Sirt3*, *Dbp1*, *Acc2*, *Pgc1α*, *Pgc1β*, and *Pparγ* in the liver tissue of *Bmal1*^*f*/*f*^ and *Bmal1*^*hep−*^^/*−*^ mice. The values are presented as mean ± SEM. *Bmal1*^*f*/*f*^ mice, n = 6; *Bmal1*^*hep−*^^/*−*^ mice, n = 6. * *p* < 0.05, ** *p* < 0.01.

The rhythmic metabolism of liver lipids is closely related to mitochondrial beta-oxidation involving molecules such as PPAR-α, CPT1, DBP1, and PGC1 [18,19]. Considering the enhanced lipolysis in *Bmal1*^*hep−*^^/*−*^ mice, we conducted assays on mitochondrial function at ZT12 (end of light phase) using mitochondria isolated from mouse livers. At the Leak state (using glutamate and malate for Leak state respiration), respiration in both groups was unaffected by *Bmal1* deletion. In the CI-linked OXPHOS and CI&II-linked OXPHOS states, *Bmal1*^*hep−*^^/*−*^ mice showed a decrease of 43.3% and 32.5%, respectively, compared to *Bmal1*^*f*/*f*^ mice, indicating a reduction in ATP production through oxidative phosphorylation. To assess the maximal capacity of the electron transfer system (ETS), uncoupler CCCP was added to reach the maximum capacity. Uncoupled respiration demonstrated that *Bmal1*^*hep−*^^/*−*^ caused a reduction of CII-linked ETS capacity by 34.6%. CIV-driven respiration was decreased by 12.1% in *Bmal1*^*hep−*^^/*−*^ mice compared to *Bmal1*^*f*/*f*^ mice (Figure 3A). However, further analysis of mitochondrial function throughout the entire process from CI-Leak to CIV state revealed that deletion of *Bmal1* led to a decline in mitochondrial function, but no statistically significant difference was observed between groups (Figure 3B). Accordingly, *Bmal1*^*hep−*^^/*−*^ mice exhibited a significant downregulation of *Acox1*, *Ppara*, and *Pgc1β* at ZT12 (Figure 3C), whereas *Cpt1α*, *Ppara*, *Dbp1*, *Pgc1α*, *Pgc1β*, and *Pparγ* were significantly downregulated at ZT0 (Figure 3D).

Considering the impact of BMAL1 on mitochondrial respiratory substrates, we utilized a Promethion Core Metabolic System to assess the respiratory exchange ratio during the 14th week of the experiment (at 22 weeks of age in mice). The *Bmal1*^*f*/*f*^ mice had a respiratory exchange ratio of 0.89643, while the *Bmal1*^*hep−*^^/*−*^ mice had a respiratory exchange ratio of 0.902038 (Supplementary Figure S2), indicating no significant difference between the two groups. These results suggested that glucose was the primary substrate for aerobic respiration and that liver-specific BMAL1 knockout does not affect the overall aerobic respiratory substrates in the body.

Taken together, these results indicated decreased hepatic mitochondrial function due to *Bmal1* deletion at both ZT0 and ZT12.

### 2.4. Profiles of Systemic Metabolism in Hepatocyte-Specific Bmal1 Knockout Mice

**Figure 4:**
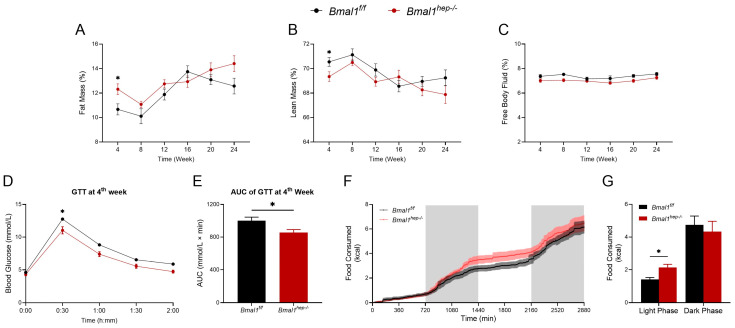
Profiles of systemic metabolism in hepatocyte-specific Bmal1 knockout mice. (**A**–**C**). Fat mass (**A**), lean mass (**B**), and free body fluid (**C**) for different time points during the experiment in *Bmal1*^*f*/*f*^ mice and *Bmal1*^*hep−*^^/*−*^ mice. (**D**,**E**). Glycemic levels during GTT (**D**) and AUC analyses of GTT (**E**) at the 4th week during the experiment in *Bmal1*^*f*/*f*^ mice and *Bmal1*^*hep−*^^/*−*^ mice. (**F**–**G**). Metabolic cage measurements of cumulative food consumption at the 14th week during the experiment in *Bmal1*^*f*/*f*^ mice and *Bmal1*^*hep−*^^/*−*^ mice. The values are presented as mean ± SEM. *Bmal1*^*f*/*f*^ mice, n = 11; *Bmal1*^*hep−*^^/*−*^ mice, n = 12. * *p* < 0.05.

At the 4th week, *Bmal1*^*hep−*^^/*−*^ mice showed a higher fat mass percentage and a lower lean mass percentage compared to the *Bmal1*^*f*/*f*^ mice (Figure 4A,B). Additionally, at the 4th week, GTT revealed that *Bmal1*^*hep−*^^/*−*^ mice demonstrated enhanced glucose tolerance compared to *Bmal1*^*f*/*f*^ mice at the 30-min mark (Figure 4D). This observation was confirmed by the area under the curve (AUC) of GTT at the 4th week (Figure 4E). Throughout the experimental period, no discernible differences were observed in body weight, food intake, and free body fluid between *Bmal1*^*f*/*f*^ and *Bmal1*^*hep−*^^/*−*^ mice (Figure 4C and Supplementary Figure S3A,B). No other differences were noted in GTT and ITT examination between *Bmal1*^*f*/*f*^ and *Bmal1*^*hep−*^^/*−*^ mice throughout the experimental period (Supplementary Figure S3C–P).

Considering the impact of BMAL1 on feeding rhythms, we utilized a Promethion Core Metabolic System to assess the impact of hepatocyte-specific *Bmal1* deletion on whole-body metabolism during the 14th week of the experiment (mice at 22 weeks of age). During the light phase (ZT0–ZT12), *Bmal1*^*hep−*^^/*−*^ mice consumed more food compared to *Bmal1*^*f*/*f*^ mice (Figure 4G). No significant differences were noted in cumulative water consumption, pedestrian distance, or intra-cage distance (Supplementary Figure S4).

## 3. Discussion

In this study, we employed *Bmal1*^*hep−*^^/*−*^ mice to provide a detailed profile of metabolism and novel insights into the impact of *Bmal1* on hepatic lipid metabolism. The main findings are summarized as follows: (1) Hepatocyte-specific knockout of *Bmal1* disrupted the expression of CCGs in the liver. (2) Under conditions of unrestricted access to food and water with a standard chow diet, liver-specific knockout mice exhibited a decrease in liver TG levels at ZT0, primarily due to enhanced lipolysis, reduced lipogenesis, and diminished lipid uptake. (3) The β-oxidation function of liver mitochondria decreased at both ZT0 and ZT12.

BMAL1 is a crucial component of the CR system, acting as a core transcriptional activator that initiates and sustains the expression of rhythmic genes within cells. This study built upon our previous research, which demonstrated that exposure to PM_2.5_ led to increased expression of *Bmal1* at ZT0/24, while key liver lipid metabolism enzymes, ACL and FAS, exhibited the most significant changes at ZT12 [12]. Consequently, we conducted a more comprehensive metabolic analysis of *Bmal1*^*hep−*^^/*−*^ mice focusing on the critical time points ZT0 and ZT12. The findings of Lamia et al. [3] also indicated that *Bmal1* function in the liver was required for systemic glucose homeostasis in a time-of-day-dependent manner. Unlike that study, our research involved mice in a state of ad libitum feeding rather than restricted feeding. Notably, in the absence of external zeitgebers such as restricted feeding, the effects of BMAL1 deletion might be driven by E26 transformation-specific (ETS) factors (another class of transcription factors regulating CR), leading to more pronounced expression rhythms in LKO mouse livers compared to control mice [20]. In this study, we confirmed the establishment of a BMAL1 conditional gene knockout mouse model at both mRNA and protein levels. In *Bmal1*^*hep−*^^/*−*^ mice, there was a significant increase in *Clock* mRNA levels at ZT12, indicating a compensatory or feedback mechanism within liver CCGs at this time point. Conversely, the expressions of *Cry2* and *Per2* were diametrically opposed to *Clock* and *Bmal1*, suggesting the primary regulatory role of BMAL1 in CCGs within the *Bmal1*^*hep−*^^/*−*^ mouse liver. REV-ERB functions as a negative regulator of BMAL1, while ROR serves as a positive regulator [21]. The rhythmic expression patterns of both genes in *Bmal1*^*hep−*^^/*−*^ mice were completely reversed compared to those in *Bmal1*^*f*/*f*^ mice, providing further confirmation of hepatic *Bmal1* deletion. Thus, peripheral BMAL1 controls the CR in the peripheral organ of liver.

Hepatic deletion of *Bmal1* induced an imbalance in the lipid metabolism by enhancing the catabolism and inhibiting the anabolism in the liver. *Bmal1* depletion has been shown to result in the inability to activate downstream PPARα, leading to a decrease in de novo lipid synthesis [22], which was consistent with the decreased expression of enzymes for lipid synthesis. In contrast, *Lpl*, the enzyme for lipolysis, increased significantly upon bmal1 ablation. Consistently, *Bmal1* deletion did protect against obesity and non-alcoholic fatty liver disease induced by a high-fat diet [23,24]. However, *Pparγ* is a target gene of BMAL1/CLOCK, and *Bmal1* deletion or *Clock* mutations in mice lead to a reduction in the expression of *Pparγ* [25]. *Pparγ* engages various transcriptional coactivators, such as PGC-1α, fostering a complex formation that binds to PPAR response elements (PPREs) within target gene promoters [26]. Our findings revealed a significant decrease in the expression of *PPARγ* and *Pgc1α* in the *Bmal1*^-/-^ mice at ZT0. Similarly, FABPs, the proteins which bind with fatty acids for further oxidation, were downregulated as well. Thus, *Bmal1* deletion may shift the cell towards the breakdown of stored lipids rather than engaging in new lipid synthesis and fatty acid oxidation. The study by Shimba et al. used a high-fat diet [24], while Chaix et al. employed a time-restricted feeding method [14]. These different dietary patterns resulted in distinct overall metabolic phenotypes. Therefore, the impact of hepatic BMAL1 deletion on lipid synthesis requires further investigation.

Although we observed no significant difference in energy production in the presence or absence of hepatic BMAL1, the diminished expression of *Bmal1* in the hepatocytes exerted a decline in mitochondrial function. BMAL1, through its circadian regulation (DBP) [27], coordinates the expression of various metabolic genes, integrating lipid metabolism (PPARs, CPT1α, and ACC2) [12], mitochondrial function (PGC1) [28], and overall energy homeostasis. Mitochondrial dynamics, particularly fission and mitophagy, as well as biogenesis, are transcriptional targets of BMAL1 [29]. Loss of BMAL1 function resulted in swollen mitochondria, diminished respiration, and elevated oxidative stress. Restoration of hepatic BMAL1 activity in high-fat-fed mice improved metabolic outcomes and rescued the morphological and metabolic defects of BMAL1-deficient mitochondria [7]. Further research is needed to understand the impact of BMAL1 loss on mitochondrial function under normal dietary conditions.

With CR disorder being recognized in metabolic diseases, BMAL1 has attracted increasing attention, and animals with global or conditional knockout of BMAL1 are ideal models for metabolism investigation. Interestingly, *Bmal1*^*hep−*^^/*−*^ mice exhibited dynamic fluctuation in metabolism, higher fat mass composition, lower lean mass composition, and improved glucose tolerance at the 4th week, but not other ages (8, 12, or 20 weeks of age). In addition, cerebral BMAL1 knockout mice exhibited reduced time on the rotarod, calorie consumption, and food and water intake [30], whereas *Bmal1*^*hep−*^^/*−*^ mice demonstrated an increased proportion of food consumption during the daytime and no significant changes in other parameters. Recently, uninterrupted rodents underwent unlimited period measurements of breathing patterns, revealing a close correlation between circadian fluctuations and O_2_ inhalation, CO_2_ exhalation, and body temperature changes, predominantly dictated by central rhythms [31]. Thus, central BMAL1 is likely the primary regulator of more comprehensive activities, while hepatic BMAL1 may be limitedly involved in the regulation of dietary behavior [14]. Additionally, more observations were shown at ZT0 but not ZT12, indicating ZT0 may be an appropriate time point for investigation into hepatic lipid metabolism in the context of CCGs. These findings provide an important basis for guiding experiment design, including animal age selection and zeitgeber time selection.

This study has certain limitations. Firstly, a more extensive sampling approach capturing additional ZT points across the circadian cycle would enhance the characterization of rhythmic variations. Secondly, the phenotype for male mice was not assessed, and it is important to recognize potential sex differences in metabolism in response to hepatic BMAL1 deletion. Addressing these limitations in future research will contribute to a more comprehensive understanding of the CR’s role in maintaining liver lipid homeostasis.

## 4. Materials and Methods

### 4.1. Reagents and Antibodies

The chemicals used in this study included EGTA (103777-10G), lactobionic acid (153516), taurine (T0625-25G), HEPES (H7523-50G), D-sucrose (84097), BSA (SRE0098-10G), pyruvate (P2256-5G), glutamate (49621-250G), malate (M1125-5G), ADP (A2754-1G), cytochrome c (C7752-50MG), succinate (S9637-100G), CCCP (C2759-250MG), rotenone (R8875-1G), antimycin A (A8674-25MG), ascorbate (PHR1279-1G), and TMPD (T3134-5G), all of which were procured from MilliporeSigma Canada Ltd. (Oakville, ON, Canada). MgCl_2_·6H_2_O (S24121-500G) was obtained from Shanghai YuanYe Biotechnology Co., Ltd (Shanghai, China). KH_2_PO_4_ (A501211-0500) was sourced from Sangon Biotech Co., Ltd (Shanghai, China). Humulin (human regular insulin) was acquired from Lilly (Indianapolis, IN, USA). Rabbit anti-mouse antibodies against BMAL1 (ab231793) were obtained from abcam Inc. (Cambridge, UK). Mouse anti-mouse antibodies against β-ACTIN (Catalog #66009-1) were obtained from Proteintech Group, Inc. (Wuhan, China).

### 4.2. Hepatocyte-Specific Bmal1 Knockout Mouse Model

All mice of the C57BL/6J background were used in this study. *Bmal1* floxed (*Bmal1*^*f*/*f*^) mice were bred by Gem Pharmatech (Gem Pharmatech Co., Ltd., NanJing, China). Hepatocyte-specific *Bmal1* knockout (*Bmal1*^*hep−*^^/*−*^) mice were generated by crossing *Bmal1*^*f*/*f*^ mice with Alb-Cre transgenic mice (Figure 1A). Seven-week-old female *Bmal1*^*f*/*f*^ and *Bmal1*^*hep−*^^/*−*^ mice were separately housed in cages with ad libitum access to a normal chow diet and water under a 12/12-h light/dark cycle at a temperature of 22 ± 2 °C. After a one-week acclimation, all mice received the same feeding regimen. ZT0 was set as 6 a.m., when the lights were turned on, and ZT12 was set as 6 p.m., when the lights were turned off. To build upon our previous findings that exposure to PM_2.5_ increases *Bmal1* expression at ZT0/24 and significantly affects the liver lipid metabolism enzymes ACL and FAS at ZT12 [12], we focused our comprehensive metabolic analysis on *Bmal1*^*hep−*^^/*−*^ mice at the critical time points of ZT0 and ZT12. At the end of the 24th week, mice were anesthetized with pentobarbital sodium (20 mg/kg, intraperitoneal injection) and liver tissues were obtained at ZT0 or ZT12 time points. The animal experiment protocol was reviewed and approved by the Animal Care and Use Committee of Zhejiang Chinese Medical University (animal use grant NO. 202107-0403).

### 4.3. Protein Extraction and Immunoblotting

At the end of the 24-week experiment (32 weeks of age in mice), an appropriate amount of liver tissue was added to the radioimmunoprecipitation assay (RIPA) lysis and centrifuged to extract the supernatant. Protein samples were prepared by quantifying the protein concentration with the bicinchoninic acid (BCA) method. Equal amounts of protein were loaded onto 8% or 10% homemade gels and then transferred to polyvinylidene fluoride (PVDF) membranes. After blocking with 5% skim milk for 90 min at room temperature, membranes were washed three times with 1 × tris-buffered saline-tween (TBST) for 10 min each time and then incubated with the corresponding primary antibodies overnight at 4 °C. After washing three times for 10 min, membranes were incubated for 1 h with the corresponding secondary antibody at ambient temperature. Immunoreactive protein levels were analyzed using a chemiluminescence imaging system (Bio-Rad, USA), and β-actin was used as an internal reference correction. Grayscale values of the protein were analyzed quantitatively with the ImageJ software (Version 1.54f, NIH, Bethesda, MD, USA).

### 4.4. RNA Extraction and Quantitative RT-PCR Analysis

At the end of the 24-week experiment (32 weeks of age in mice), RT-PCR analysis was conducted using RNA isolated from liver tissues. Total RNA extraction was performed using the Trizol reagent (TaKaRa, Shiga, Japan). Subsequently, cDNA was synthesized from the RNA using the PrimeScript RT Master Mix (TaKaRa, Shiga, Japan) following the manufacturer’s protocol. Gene expression levels were assessed using the QuantStudio Q7 system (Applied Biosystems, Carlsbad, CA, USA). The relative gene expression was calculated using the 2^−△△Ct^ method normalized to the expression of the β-actin gene. Details of the primer sequences are provided in Table 1.

### 4.5. Measurement of Hepatic TG

At the end of the 24-week experiment (32 weeks of age in mice), the experimental setup followed the guidelines provided by the TG test kits (DiaSys Diagnostics, Frankfurt, Germany) and was conducted in a 96-well plate. Approximately 100 mg of liver tissue was homogenized in the reaction buffer. Following the incubation period, absorbance readings were obtained using a Varioscan Flash microplate reader (Thermo Fisher Scientific, Waltham, MA, USA). The obtained data were subsequently normalized to the corresponding body weight.

### 4.6. Liver Oil Red O Staining

At the end of the 24-week experiment (32 weeks of age in mice), the liver tissues of mice were fixed and embedded in an optimal cutting temperature (OCT) compound (Servicebio, Wuhan, China). The embedded specimens were sectioned into 5 μm thick slices for subsequent staining with Oil Red O. A 5 μm thick slice was immersed in a freshly prepared Oil Red O working solution and kept in the dark for 8–10 min. Afterward, the slices were removed, allowed to stand for 3 s, and sequentially immersed in two containers of 60% isopropanol for differentiation for either 3 or 5 s. Subsequently, the slices were immersed in two containers of pure water for 10 s each. Hematoxylin staining was performed followed by microscopic examination of the staining effect and final sealing. The histopathological changes in the liver were observed using a Nano Zoomer S60 imaging system (Hamamatsu, Hamamatsu City, Japan). The percentage of positive areas in the liver Oil Red O staining was quantified using ImageJ software (Version 1.54f, NIH, Bethesda, MD, USA).

### 4.7. Mitochondrial Function Assays (O2K)

At ZT12 at the end of the 24-week experiment (32 weeks of age in mice), the mice were euthanized, and 2 mg of liver tissue was mechanically homogenized in a respiration medium, namely MIR05 buffer. The composition of the MIR05 buffer included 0.5 mM EGTA, 3 mM MgCl_2_·6H_2_O, 60 mM lactobionic acid, 20 mM taurine, 10 mM KH_2_PO_4_, 20 mM HEPES, 110 mM D-sucrose, and 1 g/L essentially fatty-acid-free BSA, with the pH adjusted to 7.1 at 37 °C. The liver tissue suspension was loaded into the chamber of the Oroboros 2K oxygraph system (Oroboros Instruments, Innsbruck, Austria), which was filled with the MIR05 buffer. The measurement of oxygen consumption rates proceeded with the sequential addition of substrates and specific inhibitors:

(1) Substrates of complex I (2 M pyruvate, 2 M glutamate, and 0.4 M malate, i.e., PGM) were added to maintain stable respiratory values and obtained the leak value of complex I (CI leak).
(2) Substrates of complex I-driven phosphorylating respiration (CI OXPHOS and 2.5 mM ADP) were added to maintain stable respiratory values.
(3) The intactness of the mitochondrial outer membrane was assessed by addition of exogenous 10 uM cytochrome c.
(4) Substrates of complex I- and complex II-driven phosphorylating respiration (CI+CII OXPHOS and 10 mM succinate) were added to maintain stable respiratory values.
(5) Titrating concentrations of the mitochondrial uncoupler 0.1 Mm CCCP was added to reach the maximal, uncoupled respiration (CI+CII electron transfer system, ETS).
(6) To fully inhibit complex I-driven respiration, 1 mM rotenone was added, and complex II-driven uncoupled respiration (CII electron transfer system, CII ETS) was measured.
(7) To block mitochondrial respiration at the level of complex III, 5 mM antimycin A was added.
(8) To measure cytochrome c oxidase (CIV or COX)-driven respiration, 0.8 mM ascorbate and 0.2 Mm TMPD were added.

### 4.8. Whole-Body Composition Analysis

At weeks 4, 8, 12, 16, 20, and 24 of the experiment (12, 16, 20, 24, 28, and 32 weeks of age in mice), the whole-body composition analysis was conducted. During the analysis procedure, the mice remained awake and were placed in an acrylic cylinder. The Bruker TD-NMR system (minispec LF50, Bruker, Billerica, MA, USA) was used to measure the whole-body composition. The magnetic field was set at 0.17 T and 7.5 MHz frequency pulse. The measurements of fat, lean mass, and fluid were recorded, and mice were returned to their home cage in 1 min. Data were normalized to body weight [32].

### 4.9. Evaluation of Glucose Homeostasis

At weeks 4, 8, 12, and 20 of the experiment (12, 16, 20, and 28 weeks of age in mice), the glucose homeostasis was evaluated. Mice were fasted for 12 h for intraperitoneal glucose tolerance testing (IPGTT) and were subsequently intraperitoneally injected with dextrose (2 mg/g body weight). Blood was drawn from the tail vein at 0, 30, 60, 90, and 120 min, and glucose concentration was determined using a FreeStyle glucometer (Abbott Diabetes Care Inc., Alameda, CA, USA). For insulin tolerance testing (ITT), mice were fasted for 4.5 h and then injected intraperitoneally with insulin (0.5 U/kg body weight), and blood glucose was measured at 0, 30, 60, 90, and 120 min [33]. IPGTT and ITT were performed at the 4th, 8th, 12th, and 20th weeks.

### 4.10. Metabolic Assessment

At the 14th week of the experiment (22 weeks of age in mice), the Promethion Core Metabolic System (Sable Systems International, North Las Vegas, NV, USA) was used to measure a series of metabolic parameters, including food consumption, water consumption, locomotor activity, oxygen consumption (VO_2_), carbon dioxide exhalation (VCO_2_), respiratory exchange ratio (RER), and energy expenditure (heat production). Mice were housed in individual monitoring cages of the Promethion Core Metabolic System and acclimated for 48 h before recording. Light and feeding conditions were kept the same as in the home cages. The concentrations of O_2_ and CO_2_ were measured by calculating the air entering and leaving the chambers. The respiratory exchange ratio is the ratio of CO_2_ production to O_2_ consumption. Data were normalized to body weight [34].

### 4.11. Statistical Analysis

All data were expressed as the mean ± standard error of the mean (S.E.M) unless otherwise indicated. The analyses were performed using Graphpad Prism software (Version 10.0.0, GraphPad Software, Boston, MA, USA). For the analysis, the *t*-test was used when comparing the *Bmal1*^f/f^ group with the *Bmal1*^hep−/−^ group. A *p*-value of <0.05 was considered statistically significant.

## 5. Conclusions

In summary, our research unveils the metabolic profiles of liver-specific *Bmal1* knockout in mice. Upon disrupting hepatic CR, hepatic deletion of *Bmal1* enhanced lipid catabolism and suppressed lipid anabolism and fatty acid oxidation. The present study provides direct basis for adopting liver-specific *Bmal1*-deleted animals for metabolic investigation.

## Figures and Tables

**Table 1:** Primers used for real-time PCR.

Genes	Forward Primer	Reverse Primer
*Acc1*	AAGGCTATGTGAAGGATG	CTGTCTGAAGAGGTTAGG
*Acc2*	CTTGCTTCTCTTTCTGACTTG	GGCTTCCACCTTACTGTTG
*Acox1*	CACGCACATCTTGGATGGTAGTCCG	ACGCTGGCTTCGAGTGAGGAAGTTA
*Apob*	AAACATGCAGAGCTACTTTGGAG	TTTAGGATCACTTCCTGGTCAAA
*Atgl*	GGAGACCAAGTGGAACATCTCA	AATAATGTTGGCACCTGCTTCA
*Baml1*	GCAGTGCCACTGACTACCAAGA	TCCTGGACATTGCATTGCAT
*Cd36*	TGGCCTTACTTGGGATTGG	CCAGTGTATATGTAGGCTCATCCA
*Clock*	ACCAACTGACTGGGAGTTTATG	TCTCAAGGAAGCACTGGAAAG
*Cox5a*	TGTCTGTTCCATTCGCTGCT	AGCCCATCGAAGGGAGTTTAC
*Cpt1α*	TGGCCGCATGTCAAGCCAGA	AGGAGAGCAGCACCTTCAGCGA
*Cry1*	CTGGCGTGGAAGTCATCGT	CTGTCCGCCATTGAGTTCTATG
*Cry2*	ATGTGTTCCCAAGGCTGTTC	GGTTTCTGCCCATTCAGTTC
*Dbp1*	ACGCCCTGGCGTTTGCAGAA	TGGCCACTGCTTTTCCGCCT
*Dgat1*	TGGTGTGTGGTGATGCTGATC	GCCAGGCGCTTCTCAA
*Dgat2*	AGTGGCAATGCTATCATCATCGT	TCTTCTGGACCCATCGGCCCCAGGA
*Drp1*	CAGGAATTGTTACGGTTCCCTAA	CCTGAATTAACTTGTCCCGTGA
*Fabp1*	TCAAGCTGGAAGGTGACAATAA	GTCTCCATTGAGTTCAGTCACG
*Fabp2*	TCGGTTCCTGAGGATACAAGAT	TTTGATGACTGTGGGATTGAAG
*Fabp5*	ACAGGGTTTTTGCATTCCTG	TTGGTTCTTTCGAACCTTG
*Fas*	GCGGGTTCGTGAAACTGATAA	GCAAAATGGGCCTCCTTGATA
*Hsl*	CCAGCCTGAGGGCTTACTG	CTCCATTGACTGTGACATCTCG
*Lipin*	CTATGCTGCTTTTGGGAACCG	GGACACTCCCACTTGCTTGT
*Lpl*	TTGCCCTAAGGACCCCTGAA	TTGAAGTGGCAGTTAGACACAG
*Mfn1*	ATGGCAGAAACGGTATCTCCA	GCCCTCAGTAACAAACTCCAGT
*Mfn2*	AGAACTGGACCCGGTTACCA	CACTTCGCTGATACCCCTGA
*Mtco1*	AAGCCTCCTTATTCGAGCCG	GGGGGCACCGATTATTAGGG
*Mttp*	GCTTCCGTTAAAGGTCACACA	TTTGTAGCCCACGCTGTCTT
*Ndufa1*	CAGGCCCTTGGACACATAGT	GTCCACTGCGTACATCCACA
*Ndufc1*	CGTAGTGCTGCGCTCGTTT	CTTCGACCGTGTTGAAGAGCAG
*Ndufs6*	GGGGAAAAGATCACGCATACC	CAAAACGAACCCTCCTGTAGTC
*Opa1*	TGGAAAATGGTTCGAGAGTCAG	CATTCCGTCTCTAGGTTAAAGCG
*Per1*	GCCAGGTGTCGTGATTAAATTAGTC	GGGCTTTTGAGGTCTGGATAAA
*Per2*	CAACAACCCACACACCAAAC	GCGGAATCGAATGGGAGAATA
*Pgc1α*	GAGAATGAGGCAAACTTGCTAGCG	TGCATGGTTCTGAGTGCTAAGACC
*Pgc1β*	AGAGGCACCCAGAGCGAAG	TTGTGGCATGCTGCAAATG
*Pparα*	GCGTACGGCAATGGCTITAT	GAACGGCTTCCTCAGGTTCTT
*Pparγ*	GCACTGCCTATGAGCACTTCA	CCCAAACCTGATGGCATTGTG
*Rev-erbα*	CATGGTGCTACTGTGTAAGGTGTGT	CACAGGCGTGCACTCCATAG
*Rorα*	GCGGTTGACCTCGGCATAT	ACGCTGGACTCTGCTGTTACC
*Scd1*	CTCATGGTCCTGCTGCACTT	ACGTCATTCTGGAACGCCA
*Sdhb*	TGGATCTGAATAAGTGCGGACC	GCCAGAGTATTGCCTCCGTT
*Sirt3*	CTACAGCAACCTTCAGCAGTAT	TCACATCAGCCCATATGTCTTC
*Uqcr10*	TACTCCTTGCTGTTCCGCAG	CCACAGTTTCCCCTCGTTGA
*Uqcrb*	AGGCTTCCTGAGGACCTTTA	TCCTTAGGCAAGATCTGATGC
*β-actin*	TGTGATGGTGGGAATGGGTCAGAA	TGTGGTGCCAGATCTTCTCCATGT

## Data Availability

The data that support the findings of this study are available in the methods and/or Supplementary Material of this article.

## Supplementary Materials

The following supporting information can be downloaded at: https://www.mdpi.com/article/10.3390/ijms25116070/s1.
