# Supplementary material for: Metabolic Profile and Lipid Metabolism Phenotype in Mice with Conditional Deletion of Hepatic BMAL1
Source: Int J Mol Sci. 2024 May 31;25(11):6070. doi: 10.3390/ijms25116070 (PMC11172555; doi:10.3390/ijms25116070)
Supplement: Supplementary file 1 [file ijms-25-06070-s001.zip › ijms-2978244-supplementary.pdf]

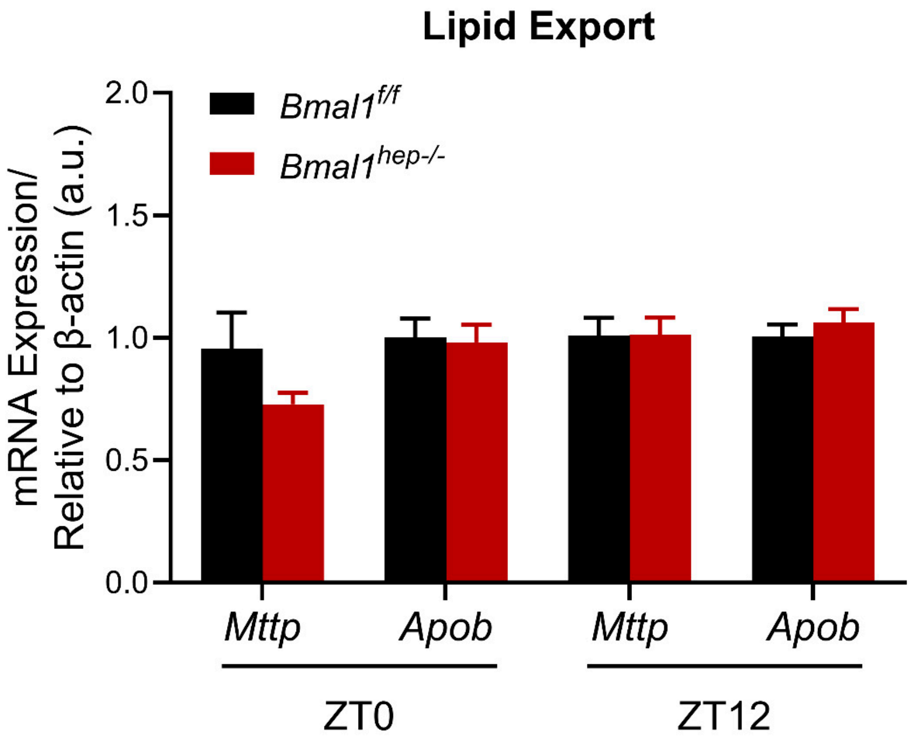

Supplementary Figure S1. The measurement of lipid lipid export genes at ZT0/12.

Bmal1$^{f/f}$ mice, n = 6; *Bmal1*$^{hep-/-}$ mice, n = 6.

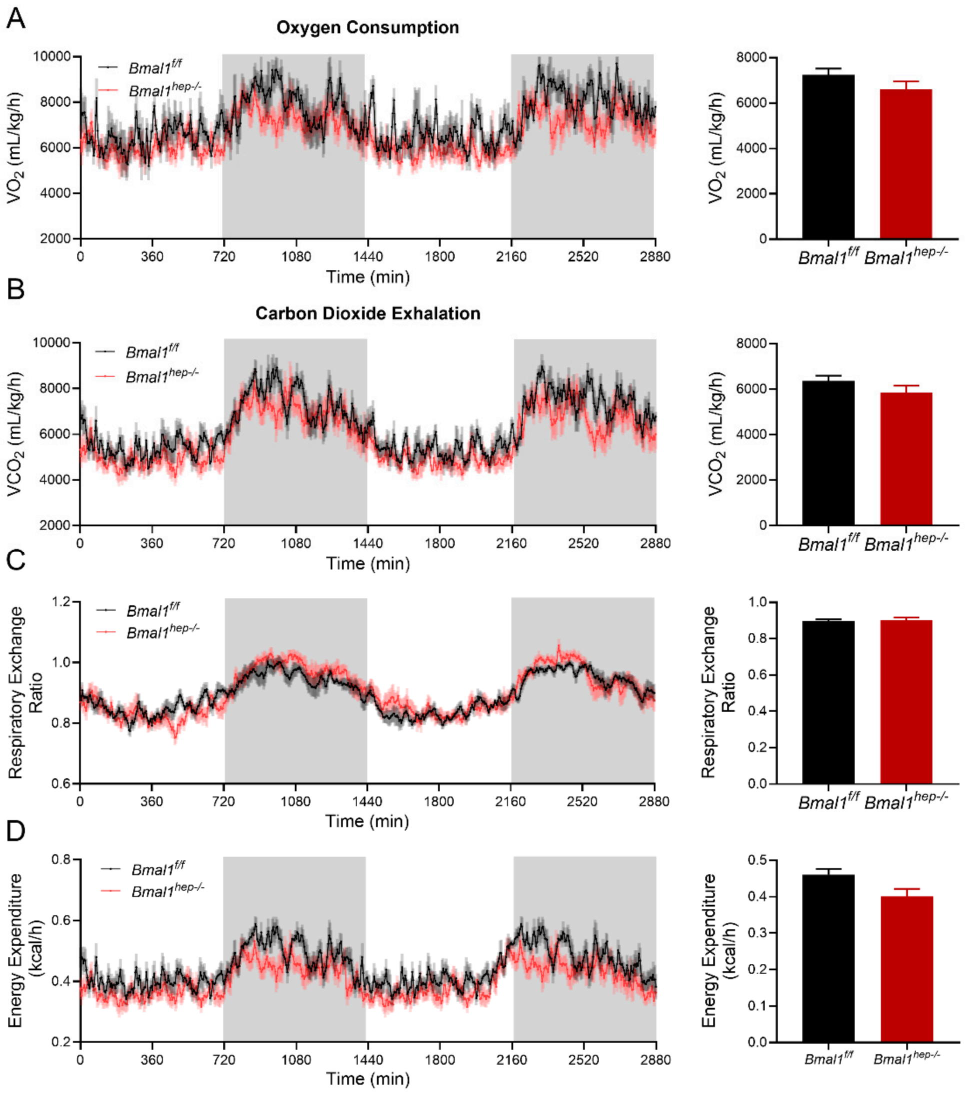

Supplementary Figure S2. Metabolic cage measurements of O_2 consumption, CO_2 consumption, respiratory exchange ratio and energy expenditure.

A. O_2 consumption and CO_2 production (B) in *Bmal1*$^{f/f}$ mice and *Bmal1*$^{hep-/-}$ mice. Respiratory exchange ratio (C) and energy expenditure (D) by Promethion system in *Bmal1*$^{f/f}$ mice and *Bmal1*$^{hep-/-}$ mice. The values are presented as mean ± SEM. *Bmal1*$^{f/f}$ mice, n = 11; *Bmal1*$^{hep-/-}$ mice, n = 12.

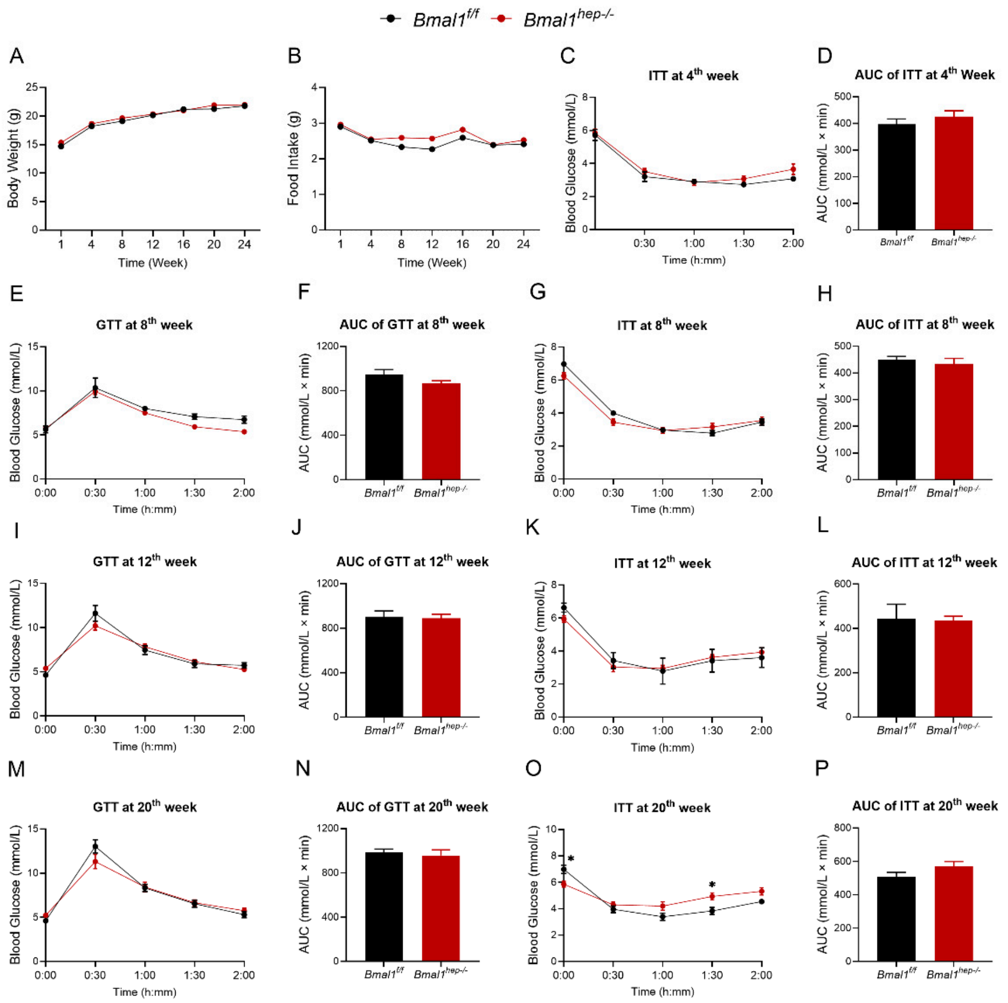

Supplementary Figure S3. Body weight, food intake, GTT, and ITT results at the 4th, 8th, 12th, and 20th weeks were analyzed along with their respective AUC.

A-B. Body weight (A), food intake (B). C-P. AUC analyses of GTT and ITT at the 4th week, 8th week, 12th week, and 20th week during the experiment in *Bmal1*$^{f/f}$ mice and *Bmal1*$^{hep-/-}$ mice. The values are presented as mean ± SEM. *Bmal1*$^{f/f}$ mice, n = 11; *Bmal1*$^{hep-/-}$ mice, n = 12.

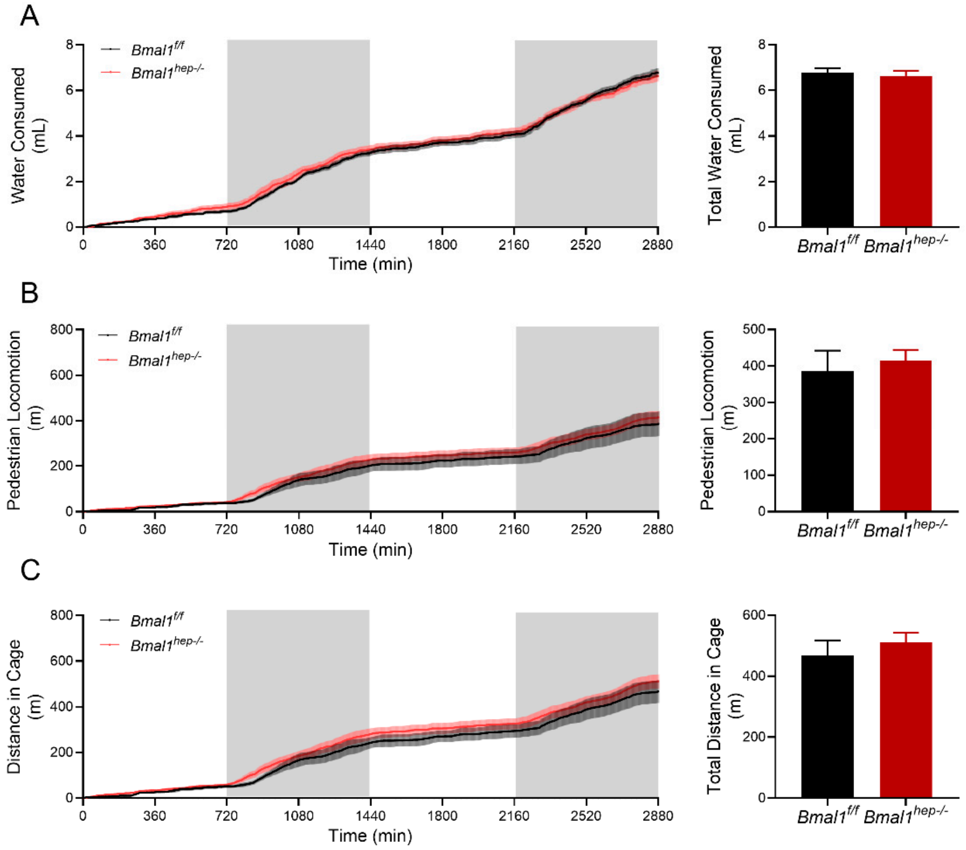

Supplementary Figure S4. Metabolic cage measurements of water consumption and intra-cage activity.

A. Metabolic cage measurements of cumulative water consumption in *Bmal1*$^{f/f}$ mice and *Bmal1*$^{hep-/-}$ mice. B. Metabolic cage measurements of cumulative pedestrian distance in *Bmal1*$^{f/f}$ mice and *Bmal1*$^{hep-/-}$ mice. C. Metabolic cage measurements of cumulative intra-cage distance in *Bmal1*$^{f/f}$ mice and *Bmal1*$^{hep-/-}$ mice. The values are presented as mean ± SEM. *Bmal1*$^{f/f}$ mice, n = 11; *Bmal1*$^{hep-/-}$ mice, n = 12. *P < 0.05.
